# Hypnotism

**Published:** 1889-10-12

**Authors:** Herbert Coryn

**Affiliations:** Trewirgie, Acre-lane, Brixton, S.W.


### EDITOR'S LETTER-BOX.

[Our Correspondents are reminded that prolixity is a great bar to publication, and that brevity of style and conciseness of statement greatly facilitate early insertion.]

### HYPNOTISM.

As you say in some interesting remarks on hypnotism in your issue of September 28, the development of this branch of science has shaken the materialism of the modern mind. But it is open to much question whether or not it is entirely desirable that public attention should be deeply turned in this direction. It appears on fairly good evidence that the Rev. Arthur Tooth has cured some cases of pronounced alcoholic craving by "suggestion" to his subjects when hypnotised that their diseased taste had departed. But of what value is such a cure? It is true that this particular expression of a sensual type of character is repressed, but the general leaning towards unearned physical luxury and pleasure, of which this vice is an index, all this basis remains. Moreover, it is clear that a conscious or unconscious suggestion towards the subsequent commission of crime would be equally faithfully followed out. Colonel Olcott is a theosophist, and I think he would as such say that those of a man's sins which are in this way removed by the will of another are only in a superficial sense cured, and that real moral stature only comes of the destruction of one's vices by one's own hand. Theosophists have revived the idea of repeated births for each individual until he has *himself* destroyed his whole lower nature, and hold, therefore, that "morality by hypnotic suggestion" is merely a convenience to a man's friends, but quite valueless in any permanent sense to the man himself.

Perhaps in a hundred years, the real authors of all great crimes will never be the mere knife-wielders of the occasion. The last will be the unfortunate tools of great hypnotising criminals sitting at home smoking, while their weak agents bear the burden and heat of the crime. We must gird up our loins so as to evolve some new or long-lost faculty of clairvoyance, in order that we may thereby be enabled to see in the thought-sphere of any man so much of his past as may be necessary to the detection of crime. Perhaps Colonel Olcott has such a faculty, and would tell us along what lines or by what laws to evolve it individually. It will be certainly the only possible weapon against oblique crime. The weaker and more hypnotisable brethren should also take steps to develop will enough for adequate resistance to suggestion. To make these experiments in mesmerism penal, except in professional hands, would, I should say, be as valueless and impotent as to make the taking of the domestic senna and rhubarb penal. We cannot limit the study of this new science, but only evolve the antidote.

Herbert Coryn (M.R.C.S., etc.).

Trewirgie, Acre-lane, Brixton, S.W.

